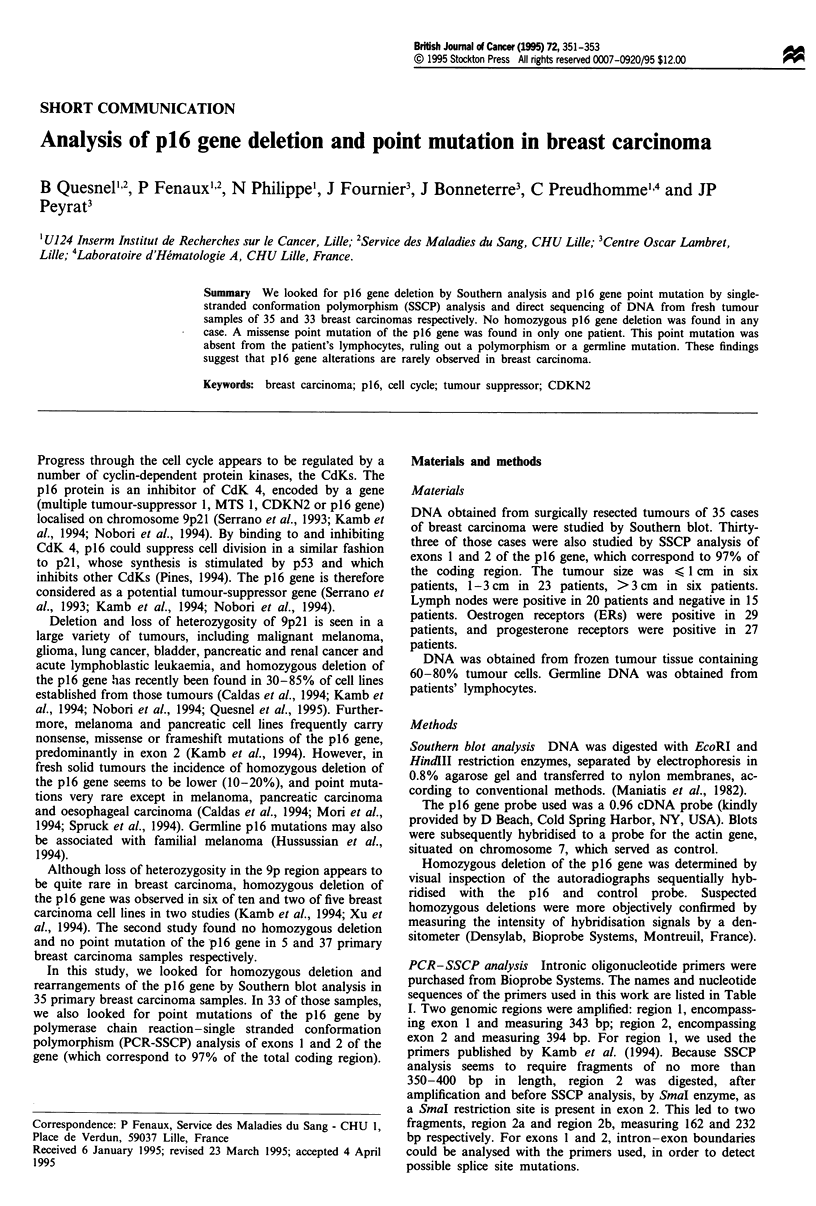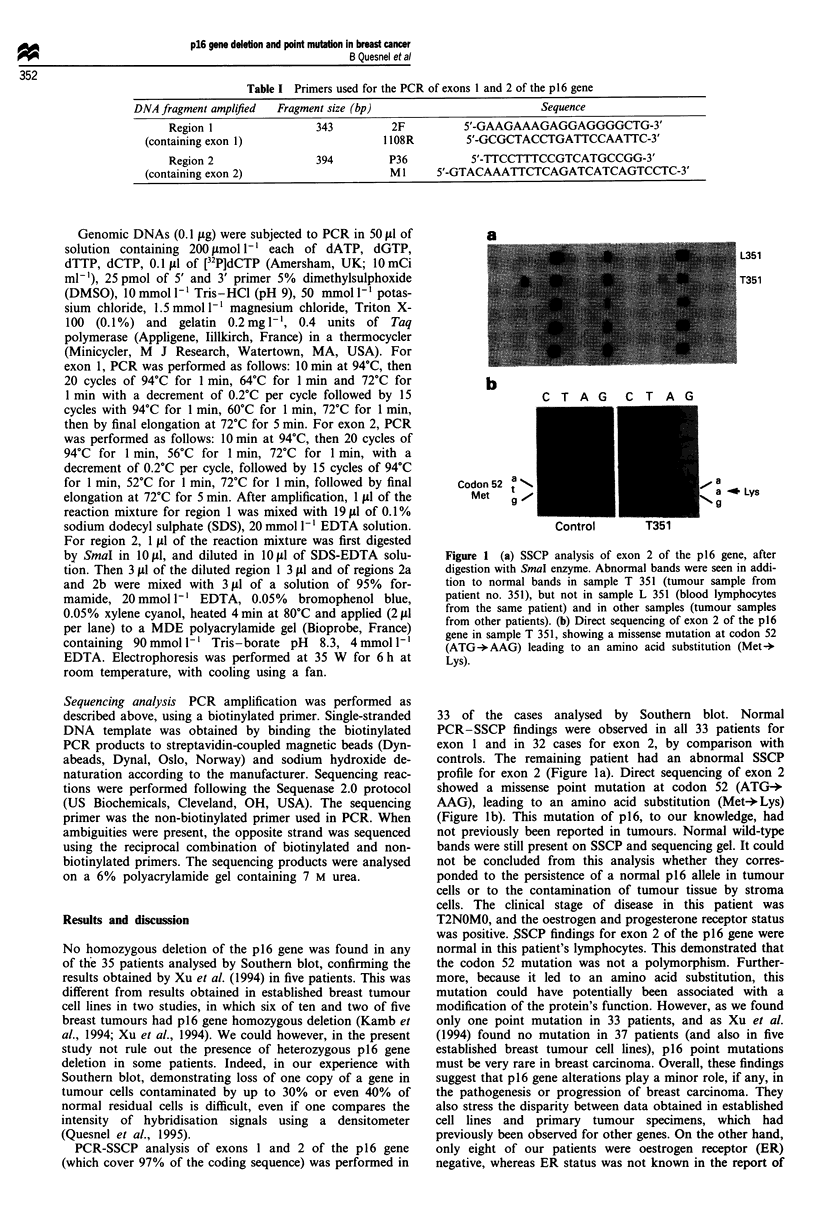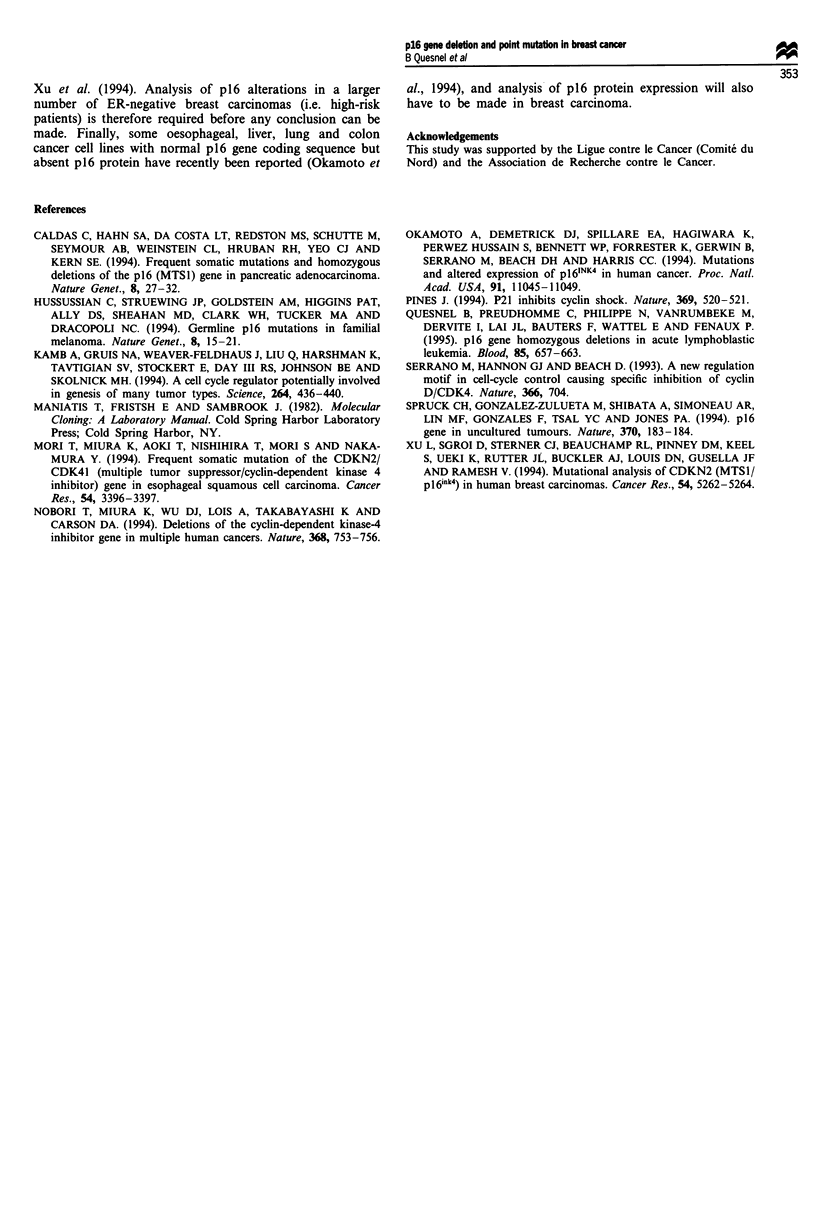# Analysis of p16 gene deletion and point mutation in breast carcinoma.

**DOI:** 10.1038/bjc.1995.337

**Published:** 1995-08

**Authors:** B. Quesnel, P. Fenaux, N. Philippe, J. Fournier, J. Bonneterre, C. Preudhomme, J. P. Peyrat

**Affiliations:** U124 Inserm Institut de Recherches sur le Cancer, Lille, France.

## Abstract

**Images:**


British Journal of Cancer (1995) 72, 351-353
© 1995 Stockton Press All rights reserved 0007-0920/95 $12.00

SHORT COMMUNICATION

### Analysis of p16 gene deletion and point mutation in breast carcinoma

B Quesnel[1,2], P Fenaux[1,2], N Philippe[1], J Fournier[3], J Bonneterre[3], C Preudhomme[1,4] and JP Peyrat[3]

[1]*U124 Inserm Institut de Recherches sur le Cancer, Lille;* [2]*Service des Maladies du Sang, CHU Lille;* [3]*Centre Oscar Lambret, Lille;* [4]*Laboratoire d'Hématologie A, CHU Lille, France.*

**Summary** We looked for p16 gene deletion by Southern analysis and p16 gene point mutation by single-stranded conformation polymorphism (SSCP) analysis and direct sequencing of DNA from fresh tumour samples of 35 and 33 breast carcinomas respectively. No homozygous p16 gene deletion was found in any case. A missense point mutation of the p16 gene was found in only one patient. This point mutation was absent from the patient's lymphocytes, ruling out a polymorphism or a germline mutation. These findings suggest that p16 gene alterations are rarely observed in breast carcinoma.

**Keywords:** breast carcinoma; p16, cell cycle; tumour suppressor; CDKN2

Progress through the cell cycle appears to be regulated by a number of cyclin-dependent protein kinases, the CdKs. The p16 protein is an inhibitor of CdK 4, encoded by a gene (multiple tumour-suppressor 1, MTS 1, CDKN2 or p16 gene) localised on chromosome 9p21 (Serrano *et al.*, 1993; Kamb *et al.*, 1994; Nobori *et al.*, 1994). By binding to and inhibiting CdK 4, p16 could suppress cell division in a similar fashion to p21, whose synthesis is stimulated by p53 and which inhibits other CdKs (Pines, 1994). The p16 gene is therefore considered as a potential tumour-suppressor gene (Serrano *et al.*, 1993; Kamb *et al.*, 1994; Nobori *et al.*, 1994).

Deletion and loss of heterozygosity of 9p21 is seen in a large variety of tumours, including malignant melanoma, glioma, lung cancer, bladder, pancreatic and renal cancer and acute lymphoblastic leukaemia, and homozygous deletion of the p16 gene has recently been found in 30-85% of cell lines established from those tumours (Caldas *et al.*, 1994; Kamb *et al.*, 1994; Nobori *et al.*, 1994; Quesnel *et al.*, 1995). Furthermore, melanoma and pancreatic cell lines frequently carry nonsense, missense or frameshift mutations of the p16 gene, predominantly in exon 2 (Kamb *et al.*, 1994). However, in fresh solid tumours the incidence of homozygous deletion of the p16 gene seems to be lower (10-20%), and point mutations very rare except in melanoma, pancreatic carcinoma and oesophageal carcinoma (Caldas *et al.*, 1994; Mori *et al.*, 1994; Spruck *et al.*, 1994). Germline p16 mutations may also be associated with familial melanoma (Hussussian *et al.*, 1994).

Although loss of heterozygosity in the 9p region appears to be quite rare in breast carcinoma, homozygous deletion of the p16 gene was observed in six of ten and two of five breast carcinoma cell lines in two studies (Kamb *et al.*, 1994; Xu *et al.*, 1994). The second study found no homozygous deletion and no point mutation of the p16 gene in 5 and 37 primary breast carcinoma samples respectively.

In this study, we looked for homozygous deletion and rearrangements of the p16 gene by Southern blot analysis in 35 primary breast carcinoma samples. In 33 of those samples, we also looked for point mutations of the p16 gene by polymerase chain reaction-single stranded conformation polymorphism (PCR-SSCP) analysis of exons 1 and 2 of the gene (which correspond to 97% of the total coding region).

Correspondence: P Fenaux, Service des Maladies du Sang - CHU 1, Place de Verdun, 59037 Lille, France
Received 6 January 1995; revised 23 March 1995; accepted 4 April 1995

### Materials and methods

### *Materials*

DNA obtained from surgically resected tumours of 35 cases of breast carcinoma were studied by Southern blot. Thirty-three of those cases were also studied by SSCP analysis of exons 1 and 2 of the p16 gene, which correspond to 97% of the coding region. The tumour size was $\leqslant 1$ cm in six patients, 1-3 cm in 23 patients, $> 3$ cm in six patients. Lymph nodes were positive in 20 patients and negative in 15 patients. Oestrogen receptors (ERs) were positive in 29 patients, and progesterone receptors were positive in 27 patients.

DNA was obtained from frozen tumour tissue containing 60-80% tumour cells. Germline DNA was obtained from patients' lymphocytes.

### *Methods*

*Southern blot analysis* DNA was digested with *Eco*RI and *Hind*III restriction enzymes, separated by electrophoresis in 0.8% agarose gel and transferred to nylon membranes, according to conventional methods. (Maniatis *et al.*, 1982).

The p16 gene probe used was a 0.96 cDNA probe (kindly provided by D Beach, Cold Spring Harbor, NY, USA). Blots were subsequently hybridised to a probe for the actin gene, situated on chromosome 7, which served as control.

Homozygous deletion of the p16 gene was determined by visual inspection of the autoradiographs sequentially hybridised with the p16 and control probe. Suspected homozygous deletions were more objectively confirmed by measuring the intensity of hybridisation signals by a densitometer (Densylab, Bioprobe Systems, Montreuil, France).

*PCR-SSCP analysis* Intronic oligonucleotide primers were purchased from Bioprobe Systems. The names and nucleotide sequences of the primers used in this work are listed in Table I. Two genomic regions were amplified: region 1, encompassing exon 1 and measuring 343 bp; region 2, encompassing exon 2 and measuring 394 bp. For region 1, we used the primers published by Kamb *et al.* (1994). Because SSCP analysis seems to require fragments of no more than 350-400 bp in length, region 2 was digested, after amplification and before SSCP analysis, by *Sma*I enzyme, as a *Sma*I restriction site is present in exon 2. This led to two fragments, region 2a and region 2b, measuring 162 and 232 bp respectively. For exons 1 and 2, intron-exon boundaries could be analysed with the primers used, in order to detect possible splice site mutations.

**Table I** Primers used for the PCR of exons 1 and 2 of the p16 gene

| *DNA fragment amplified* | *Fragment size (bp)* | | *Sequence* |
|---|---|---|---|
| Region 1 (containing exon 1) | 343 | 2F<br>1108R | 5'-GAAGAAAGAGGAGGGGCTG-3'<br>5'-GCGCTACCTGATTCCAATTC-3' |
| Region 2 (containing exon 2) | 394 | P36<br>M1 | 5'-TTCCTTTCCGTCATGCCGG-3'<br>5'-GTACAAATTCTCAGATCATCAGTCCTC-3' |

Genomic DNAs (0.1 μg) were subjected to PCR in 50 μl of solution containing 200 μmol $l^{-1}$ each of dATP, dGTP, dTTP, dCTP, 0.1 μl of [$^{32}$P]dCTP (Amersham, UK; 10 mCi $ml^{-1}$), 25 pmol of 5' and 3' primer 5% dimethylsulphoxide (DMSO), 10 mmol $l^{-1}$ Tris-HCl (pH 9), 50 mmol $l^{-1}$ potassium chloride, 1.5 mmol $l^{-1}$ magnesium chloride, Triton X-100 (0.1%) and gelatin 0.2 mg $l^{-1}$, 0.4 units of *Taq* polymerase (Appligene, Iillkirch, France) in a thermocycler (Minicycler, M J Research, Watertown, MA, USA). For exon 1, PCR was performed as follows: 10 min at 94°C, then 20 cycles of 94°C for 1 min, 64°C for 1 min and 72°C for 1 min with a decrement of 0.2°C per cycle followed by 15 cycles with 94°C for 1 min, 60°C for 1 min, 72°C for 1 min, then by final elongation at 72°C for 5 min. For exon 2, PCR was performed as follows: 10 min at 94°C, then 20 cycles of 94°C for 1 min, 56°C for 1 min, 72°C for 1 min, with a decrement of 0.2°C per cycle, followed by 15 cycles of 94°C for 1 min, 52°C for 1 min, 72°C for 1 min, followed by final elongation at 72°C for 5 min. After amplification, 1 μl of the reaction mixture for region 1 was mixed with 19 μl of 0.1% sodium dodecyl sulphate (SDS), 20 mmol $l^{-1}$ EDTA solution. For region 2, 1 μl of the reaction mixture was first digested by *Sma*I in 10 μl, and diluted in 10 μl of SDS-EDTA solution. Then 3 μl of the diluted region 1 3 μl and of regions 2a and 2b were mixed with 3 μl of a solution of 95% formamide, 20 mmol $l^{-1}$ EDTA, 0.05% bromophenol blue, 0.05% xylene cyanol, heated 4 min at 80°C and applied (2 μl per lane) to a MDE polyacrylamide gel (Bioprobe, France) containing 90 mmol $l^{-1}$ Tris-borate pH 8.3, 4 mmol $l^{-1}$ EDTA. Electrophoresis was performed at 35 W for 6 h at room temperature, with cooling using a fan.

*Sequencing analysis* PCR amplification was performed as described above, using a biotinylated primer. Single-stranded DNA template was obtained by binding the biotinylated PCR products to streptavidin-coupled magnetic beads (Dynabeads, Dynal, Oslo, Norway) and sodium hydroxide denaturation according to the manufacturer. Sequencing reactions were performed following the Sequenase 2.0 protocol (US Biochemicals, Cleveland, OH, USA). The sequencing primer was the non-biotinylated primer used in PCR. When ambiguities were present, the opposite strand was sequenced using the reciprocal combination of biotinylated and non-biotinylated primers. The sequencing products were analysed on a 6% polyacrylamide gel containing 7 M urea.

### Results and discussion

No homozygous deletion of the p16 gene was found in any of the 35 patients analysed by Southern blot, confirming the results obtained by Xu *et al.* (1994) in five patients. This was different from results obtained in established breast tumour cell lines in two studies, in which six of ten and two of five breast tumours had p16 gene homozygous deletion (Kamb *et al.*, 1994; Xu *et al.*, 1994). We could however, in the present study not rule out the presence of heterozygous p16 gene deletion in some patients. Indeed, in our experience with Southern blot, demonstrating loss of one copy of a gene in tumour cells contaminated by up to 30% or even 40% of normal residual cells is difficult, even if one compares the intensity of hybridisation signals using a densitometer (Quesnel *et al.*, 1995).

PCR-SSCP analysis of exons 1 and 2 of the p16 gene (which cover 97% of the coding sequence) was performed in

**Figure 1** (**a**) SSCP analysis of exon 2 of the p16 gene, after digestion with *Sma*I enzyme. Abnormal bands were seen in addition to normal bands in sample T 351 (tumour sample from patient no. 351), but not in sample L 351 (blood lymphocytes from the same patient) and in other samples (tumour samples from other patients). (**b**) Direct sequencing of exon 2 of the p16 gene in sample T 351, showing a missense mutation at codon 52 (ATG→AAG) leading to an amino acid substitution (Met→Lys).

33 of the cases analysed by Southern blot. Normal PCR-SSCP findings were observed in all 33 patients for exon 1 and in 32 cases for exon 2, by comparison with controls. The remaining patient had an abnormal SSCP profile for exon 2 (Figure 1a). Direct sequencing of exon 2 showed a missense point mutation at codon 52 (ATG→AAG), leading to an amino acid substitution (Met→Lys) (Figure 1b). This mutation of p16, to our knowledge, had not previously been reported in tumours. Normal wild-type bands were still present on SSCP and sequencing gel. It could not be concluded from this analysis whether they corresponded to the persistence of a normal p16 allele in tumour cells or to the contamination of tumour tissue by stroma cells. The clinical stage of disease in this patient was T2N0M0, and the oestrogen and progesterone receptor status was positive. SSCP findings for exon 2 of the p16 gene were normal in this patient's lymphocytes. This demonstrated that the codon 52 mutation was not a polymorphism. Furthermore, because it led to an amino acid substitution, this mutation could have potentially been associated with a modification of the protein's function. However, as we found only one point mutation in 33 patients, and as Xu *et al.* (1994) found no mutation in 37 patients (and also in five established breast tumour cell lines), p16 point mutations must be very rare in breast carcinoma. Overall, these findings suggest that p16 gene alterations play a minor role, if any, in the pathogenesis or progression of breast carcinoma. They also stress the disparity between data obtained in established cell lines and primary tumour specimens, which had previously been observed for other genes. On the other hand, only eight of our patients were oestrogen receptor (ER) negative, whereas ER status was not known in the report of

Xu *et al.* (1994). Analysis of p16 alterations in a larger number of ER-negative breast carcinomas (i.e. high-risk patients) is therefore required before any conclusion can be made. Finally, some oesophageal, liver, lung and colon cancer cell lines with normal p16 gene coding sequence but absent p16 protein have recently been reported (Okamoto *et al.*, 1994), and analysis of p16 protein expression will also have to be made in breast carcinoma.

**Acknowledgements**
This study was supported by the Ligue contre le Cancer (Comité du Nord) and the Association de Recherche contre le Cancer.

### References

CALDAS C, HAHN SA, DA COSTA LT, REDSTON MS, SCHUTTE M, SEYMOUR AB, WEINSTEIN CL, HRUBAN RH, YEO CJ AND KERN SE. (1994). Frequent somatic mutations and homozygous deletions of the p16 (MTS1) gene in pancreatic adenocarcinoma. *Nature Genet.*, **8**, 27-32.

HUSSUSSIAN C, STRUEWING JP, GOLDSTEIN AM, HIGGINS PAT, ALLY DS, SHEAHAN MD, CLARK WH, TUCKER MA AND DRACOPOLI NC. (1994). Germline p16 mutations in familial melanoma. *Nature Genet.*, **8**, 15-21.

KAMB A, GRUIS NA, WEAVER-FELDHAUS J, LIU Q, HARSHMAN K, TAVTIGIAN SV, STOCKERT E, DAY III RS, JOHNSON BE AND SKOLNICK MH. (1994). A cell cycle regulator potentially involved in genesis of many tumor types. *Science*, **264**, 436-440.

MANIATIS T, FRISTSH E AND SAMBROOK J. (1982). *Molecular Cloning: A Laboratory Manual.* Cold Spring Harbor Laboratory Press; Cold Spring Harbor, NY.

MORI T, MIURA K, AOKI T, NISHIHIRA T, MORI S AND NAKAMURA Y. (1994). Frequent somatic mutation of the CDKN2/CDK41 (multiple tumor suppressor/cyclin-dependent kinase 4 inhibitor) gene in esophageal squamous cell carcinoma. *Cancer Res.*, **54**, 3396-3397.

NOBORI T, MIURA K, WU DJ, LOIS A, TAKABAYASHI K AND CARSON DA. (1994). Deletions of the cyclin-dependent kinase-4 inhibitor gene in multiple human cancers. *Nature*, **368**, 753-756.

OKAMOTO A, DEMETRICK DJ, SPILLARE EA, HAGIWARA K, PERWEZ HUSSAIN S, BENNETT WP, FORRESTER K, GERWIN B, SERRANO M, BEACH DH AND HARRIS CC. (1994). Mutations and altered expression of $p16^{INK4}$ in human cancer. *Proc. Natl. Acad. USA*, **91**, 11045-11049.

PINES J. (1994). P21 inhibits cyclin shock. *Nature*, **369**, 520-521.

QUESNEL B, PREUDHOMME C, PHILIPPE N, VANRUMBEKE M, DERVITE I, LAI JL, BAUTERS F, WATTEL E AND FENAUX P. (1995). p16 gene homozygous deletions in acute lymphoblastic leukemia. *Blood*, **85**, 657-663.

SERRANO M, HANNON GJ AND BEACH D. (1993). A new regulation motif in cell-cycle control causing specific inhibition of cyclin D/CDK4. *Nature*, **366**, 704.

SPRUCK CH, GONZALEZ-ZULUETA M, SHIBATA A, SIMONEAU AR, LIN MF, GONZALES F, TSAL YC AND JONES PA. (1994). p16 gene in uncultured tumours. *Nature*, **370**, 183-184.

XU L, SGROI D, STERNER CJ, BEAUCHAMP RL, PINNEY DM, KEEL S, UEKI K, RUTTER JL, BUCKLER AJ, LOUIS DN, GUSELLA JF AND RAMESH V. (1994). Mutational analysis of CDKN2 (MTS1/$p16^{ink4}$) in human breast carcinomas. *Cancer Res.*, **54**, 5262-5264.